# Supplementary material for: Scalable, ultra-fast, and low-memory construction of compacted de Bruijn graphs with Cuttlefish 2
Source: Genome Biol. 2022 Sep 8;23:190. doi: 10.1186/s13059-022-02743-6 (PMC9454175; doi:10.1186/s13059-022-02743-6)
Supplement: Supplementary file 1 — Additional file 1. Supplementary material for “Scalable, ultra-fast, and low-memory construction of compacted de Bruijn graphs with Cuttlefish 2”. References [84–88] are cited in order in the supplementary material. [file 13059_2022_2743_MOESM1_ESM.pdf]

METHOD

Supplementary material for "Scalable, ultra-fast, and low-memory construction of compacted de Bruijn graphs with Cuttlefish 2"

Jamshed Khan[1,2], Marek Kokot[3], Sebastian Deorowicz[3] and Rob Patro[1,2*]

1 Results

1.1 Choice of frequency thresholds

The frequency threshold f_0 of k-mers ($(k+1)$-mers in case of CUTTLEFISH 2) for the algorithms when working with sequencing data was approximated so as to roughly minimize the misclassification rates of weak and solid k-mers in these experiments. This was performed based on approximate frequency distributions of the k-mer frequencies themselves, computed using the NTCARD tool [84]. The heuristic setting-policy of f_0 is inspired from observations by Zhao et al [65]: the frequency distribution of erroneous k-mers tend to diminish exponentially, whereas that of error-free k-mers typically follow a normal distribution; and the intersecting point of these density functions can be a *reasonable* choice for f_0, which we approximated with NTCARD [84]. Suppl. Fig. S1 shows some of these approximate distributions.

1.2 Compacted graph construction for sequencing data

Suppl. Table S1 contains the performance results of the evaluated tools for compacted de Bruijn graph construction from sequencing data.

Suppl. Table S2 shows the performance results on the human read set with a frequency cutoff of $f_0 = 2$.

1.3 Compacted graph construction for reference collections

Suppl. Table S3 shows the performance results of the evaluated tools for compacted de Bruijn graph construction from reference sequence collections.

1.4 Timing-profile without $(k+1)$-mer (or k-mer) enumeration

We tested the hypothesis of whether having a uniform k-mer enumerator for CUTTLEFISH 2 and BCALM 2 might significantly impact their performance difference. Suppl. Table S4 demonstrates the timing-profile of CUTTLEFISH 2 compared to BCALM 2, excluding their similar initial stage: $(k+1)$-mer and k-mer enumeration, respectively.

We find that CUTTLEFISH 2 still largely outperforms BCALM 2 in time. As for memory advantage, the BCALM 2 implementation has a "maximum memory" parameter, `-max-memory`, that could be used to restrict its memory-usage to the given argument value. In all our experiments, we set the value of `-max-memory` to the memory-usage incurred by CUTTLEFISH 2. But BCALM 2 did not strictly adhere to these limits: in both its k-mer counting and the subsequent compaction steps. [1] So replacing its k-mer enumeration step, which uses DSK [85], with KMC 3 would not necessarily constrain its memory-usage to the ones observed for CUTTLEFISH 2.

1.5 Validation of the compacted de Bruijn graphs

The validation of a compacted de Bruijn graph consists of checking three aspects of the graph: (1) completeness: whether the set of maximal unitigs contain all the k-mers from the original de Bruijn graph; (2) maximality: whether the output unitigs are actually maximal. and (3) branch-freeness: that the complete, maximal cover of the de Bruijn graph contains no paths having internal vertices than branch in the underlying de Bruijn graph.

Theoretically, the CUTTLEFISH 2 algorithm obtains all three of these criteria. Completeness is obtained trivially, and maximality and branch-freeness are obtained as per Theorem 1. We cross-checked the correctness of the actual implementation by validating the output graphs of CUTTLEFISH 2 against those of BCALM 2 and BIFROST. In doing so, we observed some small-scale differences in both the k-mer-content (completeness) and unitig-content (maximality). We provide some informal reasoning for these differences here:

[1] We verified this behavior of BCALM 2 through communicating the authors.

*Correspondence: rob@cs.umd.edu
[1] Department of Computer Science, University of Maryland, College Park, US
Full list of author information is available at the end of the article

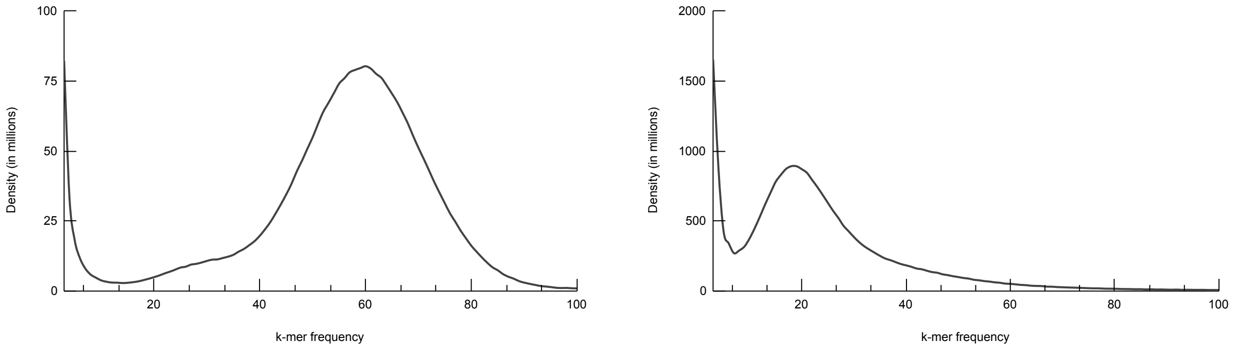

(a) **(b)**

Figure S1: Frequency distribution of k-mer abundances: (a) for the human read set NIST HG004 with $k = 27$, and (b) for the white spruce read set NCBI PRJNA83435 with $k = 55$. Densities for the frequencies 1 and 2 have been omitted from the plots, as those dwarf the other frequency densities and skew the plots drastically.

Table S1: Time-, memory-, and disk-performance results for constructing compacted de Bruijn graphs from short-read sets.

<table>
<tr><th rowspan="2">Dataset</th><th rowspan="2">k</th><th rowspan="2">Thread-count</th><th colspan="2">ABySS-Bloom-dBG</th><th>Bifrost</th><th>deGSM</th><th>BCALM 2</th><th colspan="3">Cuttlefish 2</th></tr>
<tr><th>Small-memory</th><th>Large-memory</th><th></th><th></th><th></th><th>Default memory</th><th>Match second-best memory</th><th>Un-restricted memory</th></tr>
<tr><td rowspan="4">Human</td><td rowspan="2">27</td><td>8</td><td>22h 18m
(39.3 | 0)</td><td>20h 23m
(71.3 | 0)</td><td>11h 43m
(48.5 | 0)</td><td>10h 36m
(235.8 | 737)</td><td>04h 23m
(6.7 | 344)</td><td>01h 13m
(3.2 | 209)</td><td>01h 10m
(6.2 | 209)</td><td>01h
(11.3 | 186)</td></tr>
<tr><td>16</td><td>11h 38m
(39.3 | 0)</td><td>11h 02m
(71.3 | 0)</td><td>09h 39m
(48.6 | 0)</td><td>07h 08m
(235.8 | 730)</td><td>04h 58m
(8.9 | 342)</td><td>56m
(3.3 | 209)</td><td>56m
(7.6 | 209)</td><td>51m
(11.3 | 186)</td></tr>
<tr><td rowspan="2">55</td><td>8</td><td>16h 32m
(34.0 | 0)</td><td>15h 58m
(66.0 | 0)</td><td>05h 43m
(43.8 | 0)</td><td>16h 50m
(293.2 | 1147)</td><td>04h 01m
(7.4 | 296)</td><td>02h 20m
(3.5 | 147)</td><td>01h 08m
(7.1 | 147)</td><td>01h 03m
(11.3 | 142)</td></tr>
<tr><td>16</td><td>09h 28m
(34.1 | 0)</td><td>08h 37m
(66.1 | 0)</td><td>04h 16m
(43.9 | 0)</td><td>15h 54m
(293.3 | 1147)</td><td>04h 26m
(10.5 | 293)</td><td>02h 02m
(3.7 | 147)</td><td>01h 11m
(9.5 | 147)</td><td>51m
(11.3 | 142)</td></tr>
<tr><td rowspan="2">Human RNA-seq</td><td rowspan="2">27</td><td>8</td><td>11h 47m
(33.7 | 0)</td><td>11h 22m
(65.7 | 0)</td><td>06h 04m
(7.2 | 0)</td><td>01h 35m
(87.1 | 31)</td><td>02h 58m
(3.8 | 217)</td><td>30m
(2.9 | 89)</td><td>–</td><td>18m
(80.1 | 85)</td></tr>
<tr><td>16</td><td>11h 38m
(39.3 | 0)</td><td>07h 38m
(65.7 | 0)</td><td>07h 24m
(7.2 | 0)</td><td>01h 37m
(87.2 | 31)</td><td>02h 46m
(3.9 | 217)</td><td>20m
(3.0 | 89)</td><td>–</td><td>12m
(80.1 | 85)</td></tr>
<tr><td rowspan="2">Gut microbiome</td><td>27</td><td rowspan="2">16</td><td>18h 47m
(42.0 | 0)</td><td>20h 12m
(74.0 | 0)</td><td>03h 54m
(38.1 | 0)</td><td>02h 28m
(157.2 | 362)</td><td>02h 34m
(7.7 | 165)</td><td>26m
(3.5 | 78)</td><td>23m
(6.7 | 78)</td><td>20m
(26.8 | 69)</td></tr>
<tr><td>55</td><td>1d 17h 43m
(35.9 | 0)</td><td>1d 08h 09m
(67.8 | 0)</td><td>02h 44m
(46.7 | 0)</td><td>06h 53m
(293.3 | 624)</td><td>03h 02m
(12.5 | 158)</td><td>44m
(4.0 | 52)</td><td>25m
(11.3 | 52)</td><td>20m
(69.9 | 50)</td></tr>
<tr><td rowspan="2">Soil</td><td>27</td><td rowspan="2">16</td><td>1d 18h 35m
(150.4 | 0)</td><td>14h 24m
(275.0 | 0)</td><td>15h 28m
(274.1 | 0)</td><td>1d 14h 29m
(235.8 | 3287)</td><td>19h 39m
(52.0 | 681)</td><td>02h 01m
(19.2 | 161)</td><td>02h 18m
(40.9 | 161)</td><td>01h 35m
(40.9 | 210)</td></tr>
<tr><td>55</td><td>07h 57m
(128.9 | 0)</td><td>06h 36m
(256.8 | 0)</td><td>05h 49m
(157.0 | 0)</td><td>1d 11h 05m
(293.3 | 2959)</td><td>08h 30m
(27.5 | 419)</td><td>03h 02m
(11.1 | 132)</td><td>02h 43m
(23.3 | 132)</td><td>01h 38m
(23.3 | 234)</td></tr>
<tr><td rowspan="2">White spruce</td><td>27</td><td rowspan="2">16</td><td>*</td><td>X</td><td>X</td><td>†</td><td>2d 06h 12m
(36.8 | 2171)</td><td>10h 05m
(14.0 | 1362)</td><td>07h 47m
(35.2 | 1362)</td><td>07h 13m
(204.2 | 1208)</td></tr>
<tr><td>55</td><td>*</td><td>X</td><td>X</td><td>†</td><td>2d 09h 59m
(31.6 | 1505)</td><td>10h 12m
(23.8 | 897)</td><td>10h 08m
(31.1 | 897)</td><td>07h 24m
(279.3 | 865)</td></tr>
</table>

Each cell contains the running time in wall clock format, and in parentheses: the maximum memory usage and the maximum intermediate disk-usage separated by |, in gigabytes. All the execution details and other relevant information can be found in Table 1 (see main text).

Vertex-centric versus Edge-centric de Bruijn graphs

For a given dataset, let its k-mer set be $\mathcal{V}$ and $(k+1)$-mer set be $\mathcal{E}$. Consider its vertex-centric de Bruijn graph $G_{\mathcal{V}}$ and its edge-centric de Bruijn graph $G_{\mathcal{E}}$. For ease of exposition, assume that there is no k-mer (or $(k+1)$-mer) filtering performed, and that the graphs do not have tips [2], which consist of a negligible number of vertices for real datasets. Both the graphs have the same vertex set $\mathcal{V}$.

By definition, any edge e_1 in $G_{\mathcal{E}}$ must also exist in $G_{\mathcal{V}}$. But this does not necessarily hold true in the opposite direction: some edge $e' = \{u, v\}$ in $G_{\mathcal{V}}$ could be

[2]Maximal unitigs with at least one ending side having no edge.

Table S2: Time- and memory-performance results for constructing compacted de Bruijn graphs from the human read set NIST HG004, with frequency threshold $f_0 = 2$.

k	Thread-count	ABySS-Bloom-dBG Small-memory	ABySS-Bloom-dBG Large-memory	Bifrost	deGSM	BCALM 2	Cuttlefish 2 Default memory	Cuttlefish 2 Match second-best memory	Cuttlefish 2 Un-restricted memory
27	8	Δ	1d 16h 16m (77.5)	11h 43m (48.5)	09h 34m (235.8)	06h 01m (8.9)	**01h 15m (3.9)**	01h 08m (8.6)	01h 02m (11.3)
	16	1d 14h 08m (46.9)	1d 02h 10m (77.5)	11h 02m (48.6)	08h 24m (235.8)	06h 19m (11.6)	**57m (4.1)**	52m (11.4)	49m (11.3)
55	8	Δ	1d 08h 20m (67.1)	05h 43m (43.8)	17h 23m (293.2)	05h 51m (7.7)	**02h 21m (4.1)**	01h 10m (8.5)	01h 04m (11.3)
	16	Δ	16h 29m (67.1)	04h 16m (43.9)	15h 31m (293.2)	06h 08m (10.6)	**02h 05m (4.3)**	01h (10.4)	46m (11.3)

Each cell contains the running time in wall clock format, and the maximum memory usage in gigabytes, in parentheses. Details on executing the different tool implementations can be found in Table 1 (See main text).
The best performance with respect to each metric in each row is highlighted, where only the default-memory mode is considered for Cuttlefish 2. The Δ's in the ABySS-Bloom-dBG results denote that the corresponding executions were allowed to run for at least 2 days, before being explicitly terminated.

Table S3: Time-, memory-, disk-performance results for constructing compacted de Bruijn graphs from whole-genome reference collections.

Dataset (genome count)	k	Thread-count	Bifrost	deGSM	BCALM 2	Cuttlefish 2 Default memory	Cuttlefish 2 Unrestricted memory
Human gut (30K)	27	8	06h (155.1 | 0)		10h 06m (21.5 | 473)	**01h 39m** (**15.2** | 111)	01h 39m (32.5 | 183)
		16	05h 30m (155.1 | 0)		09h 05m (22.0 | 473)	**01h 01m** (**15.5** | 111)	59m (32.5 | 183)
	55	8	08h 47m (279.2 | 0)	Δ	11h 49m (**18.6** | 708)	**04h 14m** (20.6 | 262)	03h 42m (44.4 | 480)
		16	08h 20m (279.2 | 0)		09h 45m (**19.2** | 708)	**03h 50m** (20.9 | 262)	03h 10m (44.3 | 480)
Human (100)	27	8	35h 45m (355.9 | 0)	19h 23m (235.8 | 1219)	‡	**04h 32m** (**27.7** | 311)	04h 09m (59.7 | 345)
		16	32h 14m (355.9 | 0)	14h 07m (235.8 | 1260)	‡	**03h 19m** (**28.1** | 311)	02h 49m (59.7 | 345)
	55	8	*	†	2d 23h 31m (302.9 | 2150)	**15h 08m** (**56.0** | 1288)	13h 47m (121.8 | 1332)
		16	*	†	*	**12h** (**56.2** | 1288)	11h 33m (121.8 | 1332)
Bacterial archive (661K)	27	16	X	X	‡	**16h 38m** (**48.7** | 2658)	16h 24m (104.9 | 2347)
	55				4d 10h 11m (63.3 | 2212)	**22h 44m** (**59.9** | 2047)	22h 20m (129.5 | 1974)

Each cell contains the running time in wall clock format, and in parentheses: the maximum memory usage and the maximum intermediate disk-usage separated by |, in gigabytes. All the execution details and other relevant information can be found in Table 2 (see main text).

absent in $G_\mathcal{E}$—although there exists a $(k-1)$-length overlap between the k-mers u and v, the $(k+1)$-mer $u \odot^{k-1} v$ could be absent in $\mathcal{E}$. [3] Thus $G_\mathcal{V}$ must always have an equal or greater number of edges than $G_\mathcal{E}$. As a result, $G_\mathcal{V}$ contains an equal or larger number of branching, i.e. unitig-flanking vertices than $G_\mathcal{E}$. [4] This reduces the number of maximal unitigs reported in in edge-centric de Bruijn graphs compared to vertex-centric ones.

Vertex-filtering versus Edge-filtering

Related to the above point, since the fundamental units of de Bruijn graph construction in Cuttlefish 2 are the edges, this is where error-filtering is performed prior to construction. Conversely, BCALM 2 and Bifrost take a vertex-centric approach to construction and hence filtering is performed on the vertex set. In some corner cases, where unit-abundances are very close to the selected threshold, this can lead

[3] For clarity, we are not considering the k-mer orientations.

[4] It could be possible that some of these additional edges in $G_\mathcal{V}$ connect two separate maximal unitigs into one, thus actually reducing branching vertices. The assumption that $G_\mathcal{E}$ contains no tips prevents this—there can not exist an edge $\{x, y\}$ in $G_\mathcal{V}$ such that, x and y are tip-ends in $G_\mathcal{E}$, and connect the two tips they belong to into one single maximal unitig in $G_\mathcal{V}$.

Table S4: Timing performance for constructing compacted de Bruijn graphs, excluding the initial k-mer (or (k+1)-mer) enumeration step.

Dataset	k	Thread-count	BCALM 2	CUTTLEFISH 2: Default memory	CUTTLEFISH 2: Unrestricted memory
**Short-read sets**					
Human	27	8	01h 09m	13m	11m
		16	01h 06m	09m	07m
	55	8	01h 12m	23m	17m
		16	01h 14m	15m	14m
Human RNA-seq	27	8	23m	02m	01m
		16	22m	01m	01m
Gut microbiome	27	16	01h 38m	09m	07m
	55		01h 46m	16m	13m
Soil	27	16	04h 56m	01h 17m	01h 04m
	55		05h 09m	02h 17m	01h 11m
White spruce	27	16	18h 24m	01h 30m	52m
	55		17h 17m	04h 47m	03h 15m
**Whole-genome reference collections**					
Human gut (30K)	27	8	08h 24m	01h 22m	01h 24m
		16	07h 25m	49m	46m
	55	8	09h 09m	03h 44m	03h 15m
		16	07h 34m	03h 50m	02h 45m
Human (100)	27	8	‡	03h	02h 41m
		16	‡	02h 22m	01h 31m
	55	8	2d 06h 50m	13h 17m	11h 50m
		16	*	10h 16m	09h 25m
Bacterial archive (661K)	27	16	‡	04h 16m	03h 26m
	55		1d 08h 36m	10h 50m	10h 32m

Each cell contains the running time in wall clock format, excluding the times incurred by the initial: (a) k-mer enumeration step of BCALM 2, and (b) $(k+1)$-mer enumeration step of CUTTLEFISH 2. All the execution details and other relevant information can be found in the Tables 1 and 2 (see main text).

to small-scale differences in which k-mers are filtered out prior to construction.

Consider a given threshold f_0. Any k-mer x present in the input at least f_0 times is a vertex in the vertex-centric graph. But there may not exist any $(k+1)$-mer z in the input that occurs at least f_0 times and contains x, thus x is absent as a vertex in the corresponding edge-centric graph. On the opposite direction, any k-mer y, substring of a $(k+1)$-mer z that is present in the input at least f_0 times, is a vertex in the edge-centric graph. It also implies that y has an abundance of at least f_0 in the input, and thus is also a vertex in the vertex-centric graph.

Therefore, when using a same frequency threshold f_0 for k-mers and $(k+1)$-mers, vertex-centric de Bruijn graphs must always have an equal or greater number of vertices than edge-centric ones.

1.6 Compacted de Bruijn graph properties

Suppl. Table S5 contains some notable characteristics of the original de Bruijn graphs and their compacted forms.

1.7 Maximal path cover construction

Suppl. Table S6 provides a comparison of the maximal unitig based and the maximal path cover based representations of the de Bruijn graphs.

1.8 Parallel scaling

Suppl. Fig. S2 demonstrates the timing-profile and speedup for each step of CUTTLEFISH 2, on the same setting as described in Sec. 2.7 (see main text), but with $k = 55$.

1.9 Application in associative k-mer index construction

The utility of CUTTLEFISH 2 and any compacted de Bruijn graph constructor depends upon the downstream applications for which it is used. In this proof of-concept section, we demonstrate the improvement provided by CUTTLEFISH 2 over alternative methods in a pipeline that constructs an associative k-mer index over a collection of reads or references. These indices, sometimes implicitly, form a fundamental component in various computational genomics tasks, such as in tools for variant detection and genotyping [13], RNA isoform quantification [86], large-scale sequence search [87], and k-mer abundance indexing [88].

Given a set $\mathcal{V}$ of k-mers, an associative k-mer index of $\mathcal{V}$ consists of a bijective mapping $f : \mathcal{V} \to [0, |\mathcal{V}|)$. It is different from a minimal perfect hash $h : \mathcal{V} \to [0, |\mathcal{V}|)$ in that, any alien k-mer $v \notin \mathcal{V}$ can be detected by f as absent in $\mathcal{V}$, i.e. $\forall v \notin \mathcal{V}\ f(v) = -1$; but not necessarily by h.

We investigated the overall performance difference for a pipeline that uses SSHASH [54] to index the k-mer set—represented with the de Bruijn graph—of several

Table S5: Some properties of the ordinary de Bruijn graph and its compacted form.

Dataset	k	de Bruijn graph # Vertices ($\times 10^6$)	de Bruijn graph # Edges ($\times 10^6$)	Compacted de Bruijn graph # Vertices ($\times 10^6$)	Compacted de Bruijn graph # Edges ($\times 10^6$)	Max. unitig length (kbp)
**Short-read sets**						
Human	27	2,490	2,514	58	81	21.0
	55	2,867	2,874	24	31	36.7
Human RNA-seq	27	320	309	66	55	1.1
Gut microbiome	27	2,580	2,616	205	241	5.6
	55	3,107	3,111	167	172	3.9
Soil	27	16,522	16,335	939	752	3.1
	55	9,391	9,121	432	162	2.1
White spruce	27	11,236	11,694	1,244	1,702	7.6
	55	20,536	20,725	704	894	17.0
**Whole-genome references**						
Human gut (30K)	27	13,132	13,340	569	776	92.7
	55	17,901	18,048	437	584	368.4
Human (100)	27	24,094	25,055	2,315	3,276	8.0
	55	49,220	50,039	2,122	2,941	19.2
Bacterial archive (661K)	27	42,330	42,871	1,437	1,978	114.2
	55	52,288	52,542	749	1,003	679.6

Table S6: Comparison of the maximal unitig based and the maximal path-cover based representations of de Bruijn graphs.

Dataset	k	k-mer-count	Maximal Unitigs # Unitigs	Maximal Unitigs Avg. length (bp)	Maximal Unitigs Max. length (bp)	Maximal Unitigs base/k-mer	Maximal Path-cover # Paths	Maximal Path-cover Avg. length (bp)	Maximal Path-cover Max. length (bp)	Maximal Path-cover base/k-mer
**Short-read sets**										
Roundworm	27	93,574,387	608,793	179.7	46,859	1.17	218,508	454.2	63,884	1.06
	55	96,582,016	292,444	384.3	66,206	1.16	129,203	801.5	79,500	1.07
Gut microbiome	27	2,579,749,776	204,893,577	38.6	5,633	3.07	97,631,499	52.4	6,871	1.98
	55	3,106,506,224	167,337,716	72.6	3,857	3.91	91,760,241	87.9	6,058	2.60
Human	27	2,490,358,687	57,804,370	69.1	21,012	1.60	19,811,145	151.7	21,066	1.21
	55	2,866,610,943	23,778,178	174.6	36,697	1.45	8,915,957	375.5	48,560	1.17
**Whole-genome references**										
Roundworm	27	93,471,568	527,960	203	75,221	1.15	173,552	564.6	78,941	1.05
	55	96,417,950	165,081	638.1	130,760	1.16	55,385	1,794.9	130,767	1.03
Human	27	2,431,778,046	44,459,296	80.7	29,022	1.48	14,209,926	197.1	29,034	1.15
	55	2,737,097,058	12,522,233	272.6	94,673	1.25	4,071,450	726.3	123,699	1.08
7 humans	27	2,498,416,058	54,440,059	71.9	18,424	1.57	17,507,551	168.7	30,285	1.18
	55	2,907,442,632	23,169,472	179.5	33,969	1.43	7,608,240	436.1	44,620	1.14

Given a de Bruijn graph $G(\mathcal{R}, k) = (\mathcal{V}, E)$ and a representation of it $\mathcal{P}$, the *base/k-mer* metric is computed as $\sum_{p \in \mathcal{P}} |p| \,/\, |\mathcal{V}|$, i.e. the number of nucleobase characters required in average per k-mer for the literal representation of the paths in $\mathcal{P}$ (maximal unitigs decomposition is also a path-cover). If the 2-bit/nucleobase encoding is used instead of the literal representations, then then the *bits/k-mer* requirement would be 1/4'th of the base/k-mer requirement.

datasets of various sizes. In the pipeline, we first extract the maximal path (or unitig) sequences from the graph, and then use SSHash to construct a k-mer index from these sequences. We compare the performance of this pipeline when using Cuttlefish 2 to extract the maximal path cover (or unitigs) versus using UST (or BCALM 2). Suppl. Table S7 provides a comparison of the performances.

We observe that, for intermediate or large datasets, when using UST (or BCALM 2) to extract the maximal path cover (or unitigs), the extraction of the sequences itself is both the time and the memory bottleneck. That is, indexing the extracted sequences with SSHash is both faster and more memory frugal than extracting the sequences in the first place, often by a considerable factor. On the other hand, when we use Cuttlefish 2 to extract the sequences, the time taken to extract the sequences, and therefore the time taken to construct the entire index, is reduced dramatically. Additionally, the memory usage of Cuttlefish 2 is often comparable (or in the case of the Gut microbiome reads less than) that of SSHash. Thus, replacing UST (or BCALM 2) with Cuttlefish 2 in this index construction greatly reduces the bottleneck step of associative index construction, and sometimes even shifts the bottleneck itself from the task of extracting a maximal path cover (or unitigs) of the de Bruijn graph to the task of constructing the index from these sequences.

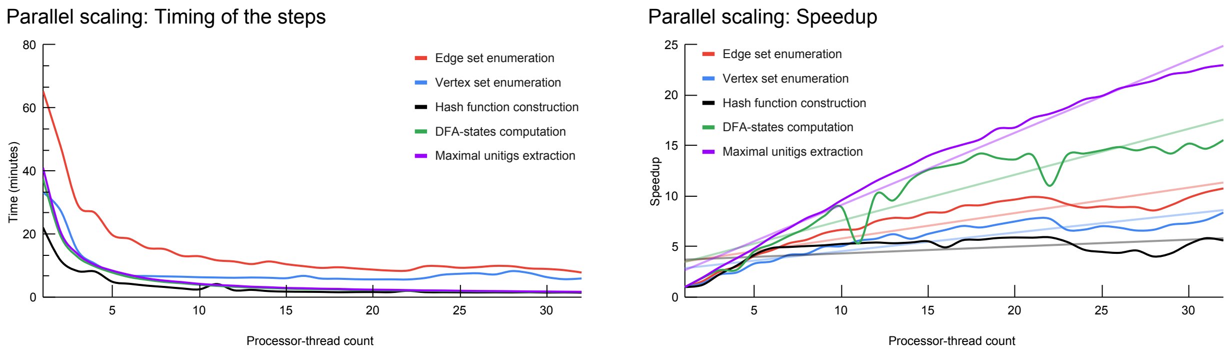

(a) Time incurred by each individual step. **(b)** Speedup of each individual step.

Figure S2: Parallel-scaling metrics for CUTTLEFISH 2 across 1–32 processor threads, using $k = 55$ on the (downsampled) human read set NIST HG004, with the frequency threshold $f_0 = 3$.

Table S7: Time- and memory-performance results for constructing an associative k-mer index using the de Bruijn graph.

Dataset	# k-mers ($\times 10^6$)					
		Maximal path cover construction		Index construction	Full pipeline	
		UST	CUTTLEFISH 2	SSHASH	UST-total	CUTTLEFISH 2-total
Human reads	2,490	04h 56m (13.1)	01h 18m (3.2)	35m (3.0)	05h 31m (13.1)	01h 53m (3.2)
Gut micro-biome reads	2,580	03h 10m (39.2)	53m (3.3)	26m (5.9)	03h 36m (39.2)	01h 20m (5.9)
Human genome ref.	2,432	01h 09m (10.3)	14m (3.2)	25m (3.0)	01h 34m (10.3)	39m (3.2)
7 human refs.	2,498	01h 49m (20.2)	18m (3.2)	25m (3.0)	02h 15m (20.2)	43m (3.2)
		Maximal unitigs construction		Index construction	Full pipeline	
		BCALM 2	CUTTLEFISH 2	SSHASH	BCALM 2-total	CUTTLEFISH 2-total
Human reads	2,490	04h 58m (8.9)	56m (3.3)	27m (4.0)	05h 25m (8.9)	01h 23m (4.0)
Human RNA-seq	320	02h 46m (3.9)	20m (3.0)	04m (2.0)	02h 50m (3.9)	24m (3.0)
Gut micro-biome reads	2,580	02h 34m (7.7)	26m (3.5)	33m (7.9)	03h 07m (7.9)	59m (7.9)

Each cell contains the running time in wall clock format, and the maximum memory usage in gigabytes, in parentheses. In all the executions, k is 27, and the maximal path cover and the maximal unitigs are the ones obtained from the experiments performed in Secs. 2.3, 2.4, and 2.5 (see main text). The dataset descriptions are also present in these sections. All the execution details and other relevant information can be found in Tables 1, 2, and 3 (see main text). The thread count used for UST and CUTTLEFISH 2 in the maximal path cover based construction is 8; for BCALM 2 and CUTTLEFISH 2 in the maximal unitigs based construction, it is 16. The SSHASH implementation is single-threaded.

1.10 Tools and execution commands

For the experiments, we used the following versions of the tools— (1) ABYSS-BLOOM-DBG from ABYSS 2.0 (v2.3.1) (2) BCALM 2 (v2.2.3) (3) BIFROST (v1.0.6.5) (4) DEGSM (v1.0) (5) PROPHASM (v0.1.1) (6) UST from ESS-COMPRESS (v2.1), (7) SSHASH (v2.1.0), and (8) CUTTLEFISH 2 (commit ID 0a049a5).

The following commands have been used in executing the tools.

- ABYSS-BLOOM-DBG:
```
abyss-bloom-dbg -b${bf_size}
-H${bf_hash_num} -j${threads}
-k${k} -kc=${min_count}
-out=${op_file} -v ${ip_files}
```
- BCALM 2:
```
bcalm -in ${ip_list}
-kmer-size ${k}
-abundance-min ${min_count}
-nb-cores ${threads}
```

```
-max-memory ${memory}
-max-disk ${disk}
-out-tmp ${temp_dir}
-out ${op_file}
```

- Bifrost:
```
Bifrost build
-${ip_type_arg} ${ip_list} -k ${k}
-t ${threads} -o ${op_file} -v
```
where `ip_type_arg` is either `r` or `s`, based on whether reference-sequences or short-reads are provided as input, respectively.
- deGSM:
```
LD_LIBRARY_PATH=${jellyfish_lib_path}
deGSM -k ${k} ${min_count_arg}
-t ${threads} -m ${memory}G
${zipped_arg} ${jellyfish_lib_path}
${op_file}.bwt ${ip_dir}
```
and
```
ubwt unipath ${op_file}.bwt
-t ${threads} -o ${op_file}
```
where `min_count_arg` is `-l ${min_count}` or empty, based on whether short-reads or reference-sequences are provided as input, respectively; and `${zipped_arg}` is `-g` if the input files are in `.gz` format, and empty otherwise.
- ProphAsm:
```
prophasm -k ${k} ${io_paths}
```
where `${io_paths}` is a concatenation of the following: `-i ${file_name} -o ${op_file}`, repeated for each different input file, with the same output file.
- UST:
```
essCompress -i ${ip_list} -k ${k}
-a ${min_count} -t ${threads}
-o ${op_dir} -u -v
```
where the `-t ${threads}` argument has been added by us to control the number of processor-cores for it to use—its default setting uses up-to all the available cores.
- SSHash:
```
cgexec -g memory:${cgroup_name}
sshash/build/build ${input} ${k}
${minimizer_len} -o ${output}
-d ${temp_dir} --verbose
```
where the `cgroup_name` is a Linux control group, set with an appropriate memory limit to restrict SSHash's reported memory usage. This is necessary due to SSHash's use of the `mmap` system call, which results in the counting of unused shared pages toward the program's memory usage, particularly in a memory-rich execution environment where the program is not under memory pressure. As such, the `time` command will report a much higher memory usage than is actually required by SSHash to run. The `cgroup` execution places a hard limit on the memory the program can use, and applies the requisite memory pressure to ensure that the reported memory usage is much closer to what is actually required for successful execution.
- Cuttlefish 2:
In the following, the `${read_or_ref_arg}` is either `read` or `ref`, based on whether reference-sequences or short-reads are provided as input, respectively.
  - Compacted de Bruijn graph construction (with default memory):
```
cuttlefish build
--${read_or_ref_arg}
-l ${ip_list} -k ${k}
-c ${min_count} -t ${threads}
-w ${temp_dir} -o ${op_prefix}
```
  - Compacted de Bruijn graph construction (with a given memory threshold):
```
cuttlefish build
--${read_or_ref_arg}
-l ${ip_list} -k ${k}
-c ${min_count} -t ${threads}
-m ${memory}
-w ${temp_dir} -o ${op_prefix}
```
  - Compacted de Bruijn graph construction (with unrestricted memory):
```
cuttlefish build
--${read_or_ref_arg}
-l ${ip_list} -k ${k}
-c ${min_count} -t ${threads}
--unrestrict-memory
-w ${temp_dir} -o ${op_prefix}
```
  - Maximal path-cover construction (with default memory):
```
cuttlefish build
--${read_or_ref_arg}
-l ${ip_list} -k ${k}
-c ${min_count} -t ${threads}
-w ${temp_dir} -o ${op_prefix}
--path-cover
```
  - Maximal path-cover construction (with unrestricted memory):
```
cuttlefish build
--${read_or_ref_arg}
-l ${ip_list} -k ${k}
-c ${min_count} -t ${threads}
--unrestrict-memory
-w ${temp_dir} -o ${op_prefix}
--path-cover
```

The scripts used to perform the experiments described in the paper are available at https://github.com/COMBINE-lab/cuttlefish_experiments.

2 Methods

2.1 Upgrades in the KMC 3 algorithm

We implemented several upgrades in the KMC 3 algorithm to tune it to the efficiency needs for CUTTLEFISH 2. Here we discuss those briefly. Although the upgrades were designed specifically for usage in CUTTLEFISH 2, those may also be suitable in other bioinformatics pipelines, and are publicly available in the KMC 3 GitHub repository (https://github.com/refresh-bio/kmc).

2.1.1 *Counting* k*-mers from existing* KMC 3 *database*

KMC 3 is updated so as to be able to count k'-mers from a k-mer database produced by another KMC 3 execution, for some $k' < k$. This allows reducing computational resources needed to determine the set of vertices, $\mathcal{V}$, as it may be directly computed from the set of edges, $\mathcal{E}$, without the need of an entire pass over all the input sequences. This is especially relevant in the case of sequencing reads. Technically, the KMC 3 API is used in the listing mode to enumerate all k-mers that are further processed as if they were reads.

2.1.2 *Estimate* k*-mer abundance histogram during the first stage in* KMC 3

This upgrade allows efficient estimation of the total number of unique k-mers present in the input during the first stage of KMC 3. The estimation is performed by our optimized implementation of the NTCARD algorithm [84].

2.1.3 *Using* KMC 3 *directly from* C++ *code with API*

A new API to use KMC 3 directly from inside some C++ code is designed for CUTTLEFISH 2, and it is usable in general. Furthermore, it is possible to set parameters for the second stage of KMC 3 based on the results of the first stage. The detailed documentation of API is available in the KMC 3 GitHub repository: https://github.com/refresh-bio/kmc/wiki/Use-the-KMC-directly-from-code-through-the-API. Combining this with the capability to estimate k-mer abundance histograms, it is possible to bound the memory-usage of the second stage of KMC 3 such that it uses at most the peak amount of memory required in the next steps of CUTTLEFISH 2.

2.1.4 *Storing* k*-mers without counts in* KMC 3 *databases*

This upgrade affects disk usage. To date, KMC 3 output required at least one byte per k-mer to store a counter. In some applications, e.g. to build the compacted de Bruijn graph without abundance estimates for the vertices, the counters are not required and can be skipped. In practice, this leads to the reduction of disk usage and, as a consequence, reduction in the total I/O costs, which in turn affects the running time.

3 Proofs

Lemma 1 *The* $(k+1)$*-mers* z *and* $\overline{z}$ *induce the same bidirected edge in a de Bruijn graph* $G(\mathcal{S}, k)$.

Proof Consider a $(k+1)$-mer z from some input string $s \in \mathcal{S}$. Let $x = \mathtt{pre}_k(z)$ and $y = \mathtt{suf}_k(z)$. Then z can be expressed as $z = x \odot^{k-1} y$.

z induces an edge between the vertices $\widehat{x}$ and $\widehat{y}$. It is incident to the $\mathtt{back}$ of $\widehat{x}$ when $\widehat{x} = x$ holds, and is incident to the $\mathtt{front}$ when $\widehat{x} = \overline{x}$ (see Sec. 3.2, main text).

z's reverse complement is $\overline{z} = \overline{y} \odot^{k-1} \overline{x}$, and it induces an edge between $\widehat{y}$ and $\widehat{x}$. It is incident to the $\mathtt{front}$ of $\widehat{x}$ if $\widehat{x} = \overline{x}$ holds, and is incident to the $\mathtt{back}$ if $\widehat{x} = x$—the same side as that of z's edge.

It can be proven likewise that the edges are incident to the same side of $\widehat{y}$. Therefore, z and $\overline{z}$ induce edges between the same vertex-pair $\{\widehat{x}, \widehat{y}\}$, incident to the same sides—inducing the same bidirected edge. ∎

Lemma 2 *A side of a vertex can have at most* $|\Sigma|$ *distinct edges in a de Bruijn graph* $G(\mathcal{S}, k)$.

Proof Consider a vertex $\widehat{v}$ in $G(\mathcal{S}, k)$. WLOG, we prove the claim for the $\mathtt{back}$ of $\widehat{v}$.

An edge e connected to $\widehat{v}$ and induced by a $(k+1)$-mer z is incident to $\widehat{v}$'s $\mathtt{back}$ iff: (1) $\mathtt{pre}_k(z) = \widehat{v}$; or (2) $\mathtt{suf}_k(z) = \overline{\widehat{v}}$ (see Sec. 3.2, main text). For case (1), the possible z's form the set $\mathcal{E}_1 = \{\widehat{v} \cdot c : c \in \Sigma\}$. For case (2), the set is $\mathcal{E}_2 = \{c' \cdot \overline{\widehat{v}} : c' \in \Sigma\}$, which is the same as $\{\overline{c} \cdot \overline{\widehat{v}} : c \in \Sigma\}$, letting $c' = \overline{c}$ [5].

As per Lemma 1, the $(k+1)$-mers $c \cdot \widehat{v}$ and $\overline{\widehat{v}} \cdot \overline{c}$ induce the same bidirected edge, where $c \in \Sigma$. Thus $\mathcal{E}_1$ and $\mathcal{E}_2$ induce the same set of edges. Therefore, the $\mathtt{back}$ of $\widehat{v}$ can have at most $|\mathcal{E}_1| = |\mathcal{E}_2| = |\Sigma|$ distinct edges. ∎

Lemma 3 *A vertex* $\widehat{v}$ *is noted to be a flanking vertex in a de Bruijn graph* $G(\mathcal{S}, k)$ *iff it is an endpoint of a maximal unitig.*

Proof Let $\mathcal{C}_v$ be the state-class of $\widehat{v}$'s automaton and p be the maximal unitig containing $\widehat{v}$. The term *branching* in the proof means connecting to multiple distinct edges.

First, assume that $\widehat{v}$ is marked as a flanking vertex. We prove that $\widehat{v}$ is an endpoint of p. As per the definition of flanking vertices (see Sec. 3.3.8, main text), either of the following holds:

[5] As per our definitions, the set Σ of symbols is closed under complementing.

1. $\mathcal{C}_v$ is not *unique-front unique-back*. Then from Corollary 1, $\hat{v}$ has at least one side s_v with either 0 or > 1 distinct edges. It is not possible to extend p through s_v—either there is no edge, or the addition introduces an internal branching vertex $\hat{v}$ in p.
2. $\mathcal{C}_v$ is *unique-front unique-back*, and a side of it, s_v, is connected to a branching side s_u of a vertex $\hat{u}$. Then p can not be extended through s_v, because the extension includes s_u as an internal side to p, which is branching.

In either case, $\hat{v}$ is an endpoint of p.

Now assume that $\hat{v}$ is an endpoint of p. We prove that $\hat{v}$ is marked as a flanking vertex. Based on the adjacencies of $\hat{v}$, either of the following holds:

1. $\hat{v}$ has at least one side s_v that is either empty or branching. From Corollary 1, C_v is not *unique-front unique-back.*
2. $\hat{v}$ has one unique edge at each side. Say that its side s_v restricts p from extending farther, and s_v connects to the side s_u of a vertex $\hat{u}$. The definition of unitigs implies that s_u must be branching. This in turn implies from Corollary 1 that $\hat{u}$'s automaton's state is from the state-class: (i) either *fuzzy-front fuzzy-back*; or (ii) *fuzzy-front unique-back*, in which case s_u is $\mathtt{front}$; or (iii) *unique-front fuzzy-back*, in which case s_u is $\mathtt{back}$.

In either case, $\hat{v}$ fulfills the conditions for being a flanking vertex. ■

Corollary 1 *For the automaton M_v of a vertex $\hat{v}$ in a de Bruijn graph $G(\mathcal{S}, k)$, applying δ on M_v with all the incident edges of $\hat{v}$ (in any order) transitions its state from q_0 to q_v belonging to the state-class C_v, such that C_v is:*

1. *fuzzy-front fuzzy-back, iff $\hat{v}$ does not have exactly one unique edge at any of its sides*
2. *fuzzy-front unique-back, iff $\hat{v}$ has exactly one unique edge only at its* $\mathtt{back}$
3. *unique-front fuzzy-back, iff $\hat{v}$ has exactly one unique edge only at its* $\mathtt{front}$
4. *unique-front unique-back, iff $\hat{v}$ has exactly one unique edge at each of its sides.*

Proof The proof is trivial from the definition of the transition function δ, illustrated in detail in Fig. 4 (see main text). ■

Theorem 1 CUTTLEFISH $2(\mathcal{R}, k, f_0)$ *is correct.*

Proof Following from Corollary 1, the COMPUTE-AUTOMATON-STATES algorithm correctly computes the state-classes of all the automata. Besides, CUTTLEFISH 2's modeling scheme of a vertex $\hat{v}$ with an automaton M_v ensures that if a side s_v has a unique incident edge e, an encoding of e is preserved in M_v's state q_v, observable from the illustration of δ in Fig. 4 (see main text). Hence, all the internal edges of the maximal unitigs are retained within the states.

For some vertex $\hat{v} \in \mathcal{V}$, let p be the maximal unitig containing $\hat{v}$, and $p = (\hat{v}_0, e_1, \hat{v}_1, \dots, e_\ell, \hat{v}_\ell)$, with $\hat{v} = \hat{v}_i$. The Extract-Maximal-Unitigs algorithm starts two walks w_b and w_f from $\hat{v}_i$, respectively through its $\mathtt{back}$ and $\mathtt{front}$, using the algorithm Walk-Maximal-Unitig. WLOG, assume that e_i and e_{i+1} are incident to the $\mathtt{front}$ and to the $\mathtt{back}$ of $\hat{v}_i$, respectively. Also, let $p_f = (\hat{v}_0, e_1, \dots, e_i, \hat{v}_i)$, and $p_b = (v_i, e_{i+1}, \dots, e_\ell, \hat{v}_\ell)$. First consider the case that $|p_b| > 1$, so $i < \ell$. Since the $\mathtt{back}$ of $\hat{v}_i$ is internal to p, it only has the edge e_{i+1}, encoded in the automaton M_v's state. So w_b must exit $\hat{v}_i$ using e_{i+1}, entering $\hat{v}_{i+1}$. Now each $\hat{v}_j$ $(i < j < \ell)$ being an internal vertex to p, it only has the unique edges e_j and e_{j+1}, one per each side. So w_b enters each $\hat{v}_j$ with e_j and exits it with e_{j+1}, thus continuing on. And it is not possible for w_b to deviate off p without reaching $\hat{v}_\ell$, where it terminates finding $\hat{v}_\ell$ to be flanking. Besides, early termination at some internal $\hat{v}_j$ $(i < j < \ell)$ does not occur either, as Lemma 3 implies that no internal vertex is flanking. Thus w_b traverses p_b in its entirety. For the case when $|p_b| = 1$, w_b terminates immediately finding $\hat{v}_i$ to be flanking. Thus in either case, w_b extracts p_b correctly.

By symmetry, w_f extracts p_f correctly. Therefore p is correctly constructed by joining p_f and p_b at $\hat{v}_i$.

Since each $v \in \mathcal{V}$ is processed in this manner to compute its containing maximal unitig, CUTTLEFISH 2 correctly extracts the entire set of maximal unitigs of $G(\mathcal{R}, k)$. ■
