# Supplementary material for: Scalable, ultra-fast, and low-memory construction of compacted de Bruijn graphs with Cuttlefish 2
Source: Genome Biol. 2022 Sep 8;23:190. doi: 10.1186/s13059-022-02743-6 (PMC9454175; doi:10.1186/s13059-022-02743-6)
Supplement: Supplementary file 2 — Additional file 2. Review history. [file 13059_2022_2743_MOESM2_ESM.docx]

**1^st^ round**

**Reviewer 1**

In this manuscript, the authors described a novel method, cuttlefish2, for constructing compacted de bruijn graphs both time and memory efficiently. The authors compared with other similar tools on both small and large datasets, and the proposed method cuttlefish2 outperforms all other tools in both time and memory costs. Overall, the paper is written and organized well. I have the following concerns:

1. The authors introduced several potential applications like de novo assembly from long reads, SV identification and genotyping, and pan-genome analysis. The current direct output is the maximal unitigs, and it's unclear how to extend to potential applications. Also, it's better to have a clear definition of potential direct applications for the proposed method rather than for general de bruijn graphs. For example, from my understanding, the current strategy to cut reads to kmers for assembly does not show clear advantages over overlap-based assemblies on long reads. And on pan-genome analysis, what's the advantages over tools like vg and minigraph? Maybe this is out of scope for the method itself, but one or some applications of the proposed approach will convince the potential users.

2. It's unclear for me whether the advantage over other tools in memory consumption comes from cuttlefish2 or from kmc3? It is known that kmc3 is memory efficient from kmer counting, and if other tools also adapt kmc3 for this step, will cuttlefish2 still outperform other tools?

3. I tried to install cuttlefish2 on a slurm cluster, and failed in the both ways:
Install from source:
collect2: error: ld returned 1 exit status
make[3]: *** [kmc] Error 1
make[2]: *** [prj_kmc-prefix/src/prj_kmc-stamp/prj_kmc-build] Error 2
make[1]: *** [CMakeFiles/prj_kmc.dir/all] Error 2
make: *** [all] Error 2

Install from conda:
conda install -c bioconda cuttlefish (this step run successfully)
cuttlefish build -s reads.fq.gz -k 3 -t 4 -o cdbg -w temp/ --read -c 1
Option 'read' does not exist (got this error message)

**Reviewer 2**

This is a well-written paper about cuttlefish 2, a high quality new tool for constructing compact De Bruijn Graphs (cDBG). Cuttlefish 2 is presented as being consistently faster and lower memory than the other major cDBG construction tools. Conveniently, the algorithmic underpinnings seem to be much simpler than the other cDBG tools as well, making this a nice theoretical simplification of the problem, likely due to the availability of good minimal perfect hashing functions.

The main bottleneck in compacting DBGs is the memory to store the DBG and/or remove errors. BCALM 2, one of the main competitors against which they benchmark, splits the graph into bins and processes each bin separately; in contrast, cuttlefish splits the data into bins in the k-mer counting step (using kmc3) and loads the DBG vertices only when extracting the unitigs. Here a limitation of cuttlefish is that it must still load all the k-mers into memory (and thus can provide no upper bound on memory consumption), although it is extremely memory efficient because it uses a MPHF that stores k-mers in 1.2 bytes per k-mer,and errors are filtered during the kmer counting step by KMC3

The paper is overall excellent and cuttlefish is likely to be a well-used software tool, too.

We had several concerns about the paper that could be addressed by adjusting the text and/or a few new computational experiments.

First, how were the cDBGs validated? We've run into minor variations in k-mer content (it seems to be mostly around repeat structures and other "annoying" graph features) between BCALM and bifrost, for example. So we're curious about how cuttlefish's results were compared and validated. It would be good to have a sentence or two about this.

Second, on page 5 line 33, the authors state "BCALM 2 also has a maximum-memory option, which we set to the best memory usage obtained from the rest of the algorithms." Is there a reason here not to use the max memory setting to that actually used by cuttlefish, for direct comparison? It should be straightforward to show that bcalm can work within the same memory limits as cuttlefish, but slower.

Third, we think an experiment using an RNA-seq dataset might be relevant as it has a different k-mer spectra, and removing errors with a minimum abundance threshold is not as effective as with DNA samples.

Minor comments:

* command lines are provided in the supplementary materials but without the actual parameter values. The authors should make their benchmarking scripts available.
* there is a typo in Figure 2 and Supplementary Figure 2: 'contigs extraction' should be 'unitigs extraction'.
* on page 11 column2, Sv is defined as the side of vertex v, while S is defined as a string, and S1 is the first character in the string. We recommend using a different letter for the side of vertex.
* on page 12 line 59, the reference [75] is misleading. The text is talking about piecewise graph traversal, but the reference seems to be about graph traversal techniques?
* the authors described how they calculated f0 (minimum abundance) in the supplementary material but they didn't provide the actual values used by each experiment.
* in table 2 and 3, we noticed that CUTTLEFISH2 unrestricted memory mode uses two or more times the memory used in the default memory parameters, while the run time is approximately the same. So, what would motivate a user to go for the unrestricted memory mode?

**Authors’ response**

We would like to thank all the reviewers for their constructive feedback, which we consider to have improved the manuscript and clarified the exposition of the work.

**Reviewer 1**

In this manuscript, the authors described a novel method, cuttlefish2, for constructing compacted de bruijn graphs both time and memory efficiently. The authors compared with other similar tools on both small and large datasets, and the proposed method cuttlefish2 outperforms all other tools in both time and memory costs. Overall, the paper is written and organized well.

We thank the reviewer for their appreciation of the paper.

1. The authors introduced several potential applications like de novo assembly from long reads, SV identification and genotyping, and pan-genome analysis. The current direct output is the maximal unitigs, and it’s unclear how to extend to potential applications. Also, it’s better to have a clear definition of potential direct applications for the proposed method rather than for general de bruijn graphs. For example, from my understanding, the current strategy to cut reads to kmers for assembly does not show clear advantages over overlap-based assemblies on long reads. And on pan-genome analysis, what’s the advantages over tools like vg and minigraph? Maybe this is out of scope for the method itself, but one or some applications of the proposed approach will convince the potential users.

We thank the reviewer for this observation. We agree that, ultimately, the utility of Cuttlefish 2 and, in fact, any compacted de Bruijn graph constructor, will depend upon the downstream applications for which it is used. While we have enumerated many use cases of the compacted de Bruijn graph in the current manuscript (in Sec. 1 Background), new applications are regularly being proposed by the community. While we believe that a through investigation of the impact that Cuttlefish 2 will have on the potential pipelines where it may be adopted is outside the scope of the current manuscript, we have added a new section: Suppl. Sec. 1.8 (Application in associative k-mer index construction), referring to it from the main text, at Sec. Conclusion. This section demonstrates the improvement provided by Cuttlefish 2 over alternative methods in a pipeline that builds an associative k-mer index over a collection of reads or references. We anticipate that Cuttlefish 2 will provide similar benefits in other applications where the community chooses to apply it.

2. It’s unclear for me whether the advantage over other tools in memory consumption comes from cuttlefish2 or from kmc3? It is known that kmc3 is memory efficient from kmer counting, and if other tools also adapt kmc3 for this step, will cuttlefish2 still outperform other tools?

The memory advantage comes from both directions: Cuttlefish 2 has a fixed required memory-budget—9.7 bits/k-mer, which has been shown to outperform the rest of the tested methods; and KMC 3 can operate within a fixed memory-budget, which we specifically set to 9.7 x k-mer_count bits beforehand. The k-mer count is approximated within KMC 3, an extension implemented by us as a part of this work: please check Suppl. Sec. 2.1.2.

For the inquiry about using KMC 3 as part of the other tools, we had already tested this hypothesis by providing comparison results (in Suppl. Sec. 1.4) against BCALM 2, our closest competitor. Specifically, we compare the performance of BCALM 2 vs Cuttlefish 2 excluding their initial step of k-mer (or (k + 1)-mer for Cuttlefish 2) enumeration, and show that Cuttlefish 2 still outperforms BCALM 2 largely in time. The results are presented in Suppl. Table 4. As for memory advantage, BCALM 2 has a “maximum memory” parameter, -max-memory, that could be used to restrict its memory-usage to the given argument value. In all our experiments, we set the value of -max-memory to the memory-usage incurred by Cuttlefish 2. But BCALM 2 did not strictly adhere to these limits: in both its k-mer counting and the subsequent compaction steps. So replacing its k-mer counting step, which uses DSK [1], with KMC 3 would not necessarily constrain its memory-usage to the ones observed for Cuttlefish 2. This is particularly likely to be the case as DSK (including the optimized version used in BCALM 2), like KMC 3, adopts an external memory algorithm for counting k-mers. As a side note, we have also confirmed this behavior of the BCALM 2 implementation, i.e. not conforming to the provided memory bound, with the BCALM 2 authors.

For the other tools tested: ABySS-Bloom-dBG and Bifrost do not use explicit k-mer counting as part of their pipeline, so the argument is inapplicable for them. deGSM uses Jellyfish to enumerate (k + 2)-mers and k-mers, and could benefit from using KMC 3 for their memory-usage. But the rest of the process in deGSM is quite heavy in disk-IO operations, and from our observations in using deGSM in the experiments, it would not gain a significant advantage in time using KMC 3.

We have added a couple of sentences to the Suppl. Sec. 1.3 (Timing-profile without (k + 1)-mer (or k-mer) enumeration), summarizing the above.

3. I tried to install cuttlefish2 on a slurm cluster, and failed in the both ways: Install from source:

collect2: error: ld returned 1 exit status

make[3]: *** [kmc] Error 1

make[2]: *** [prj_kmc-prefix/src/prj_kmc-stamp/prj_kmc-build] Error 2

make[1]: *** [CMakeFiles/prj_kmc.dir/all] Error 2

make: *** [all] Error 2

There is a possibility that the source-compile bug in slurm originated in the compilation of the Pythonwrapper of the KMC API, which is not needed in our case. We updated the build procedure to skip this, which might solve the problem.

Install from conda:

conda install -c bioconda cuttlefish (this step run successfully)

cuttlefish build -s reads.fq.gz -k 3 -t 4 -o cdbg -w temp/ --read -c 1

Option 'read' does not exist (got this error message)

We believe that Bioconda provided the reviewer with the Cuttlefish 1.0 package. This could be due to a host of reasons: might be the lack of a fresh environment, or an explicit request for version 2.0 might be required, etc. For example, the Cuttlefish 2.0 package can be explicitly requested as follows:

conda create --name cuttlefish

conda activate cuttlefish

conda install -c bioconda cuttlefish=2.0

**Reviewer 2**

This is a well-written paper about cuttlefish 2, a high quality new tool for constructing compact De Bruijn Graphs (cDBG). Cuttlefish 2 is presented as being consistently faster and lower memory than the other major cDBG construction tools. Conveniently, the algorithmic underpinnings seem to be much simpler than the other cDBG tools as well, making this a nice theoretical simplification of the problem, likely due to the availability of good minimal perfect hashing functions.

We thank the reviewer for appreciating the quality of the tool and its theoretical underpinnings.

The main bottleneck in compacting DBGs is the memory to store the DBG and/or remove errors. BCALM 2, one of the main competitors against which they benchmark, splits the graph into bins and processes each bin separately; in contrast, cuttlefish splits the data into bins in the k-mer counting step (using kmc3) and loads the DBG vertices only when extracting the unitigs. Here a limitation of cuttlefish is that it must still load all the k-mers into memory (and thus can provide no upper bound on memory consumption), although it is extremely memory efficient because it uses a MPHF that stores k-mers in 1.2 bytes per k-mer,and errors are filtered during the kmer counting step by KMC3

We require two pieces of information for each k-mer: (1) a minimal perfect hash function over the set of the filtered unique k-mers (3.7 bits/k-mer), and (2) a state for the k-mer’s automaton (6 bits/k-mer). This means that an upper bound for the memory usage is 9.7n bits ≈ 1.2n bytes, when the graph has n vertices. Although it is true that the memory usage cannot be bounded by some constant in a straightforward manner, through binning of the graph vertices.

The paper is overall excellent and cuttlefish is likely to be a well-used software tool, too.

We thank the reviewer for their appreciation of the paper and the tool.

We had several concerns about the paper that could be addressed by adjusting the text and/or a few new computational experiments.

First, how were the cDBGs validated? We’ve run into minor variations in k-mer content (it seems to be mostly around repeat structures and other “annoying” graph features) between BCALM and bifrost, for example. So we’re curious about how cuttlefish’s results were compared and validated. It would be good to have a sentence or two about this.

The reviewer raises an excellent point here, as the validation of the compacted de Bruijn graph, especially on large scale inputs, can be particularly difficult. One can view three aspects in which the compacted dBG can be validated; completeness

— does the set of unitigs contain all of the expected k-mers? maximality

— have all unitigs been maximally compacted? and branch-freeness

— does each unitig contain only non- branching vertices as internal vertices? Theoretically, the Cuttlefish algorithm obtains all these, and so the main question is if this theory translates into practice (i.e. if there are bugs in the code).

In our validation we compared the results of Cuttlefish 2 to that of Bifrost and BCALM 2 and observed some small differences in the unitig and k-mer content. Namely, the number of unitigs and number of k-mers from the Cuttlefish outputs have always been equal or lower than those from of Bifrost and BCALM 2.

For de Bruijn graphs constructed from reference sequences, i.e. when using frequency threshold of 1, the k-mer content among all the outputs has always been the same. On the other hand, when constructed from read sets, the k-mer count in Cuttlefish 2 is always a bit lower.

We have provided some informal reasoning for the small-scale differences between the unitig and the k-mer counts by adding a new Suppl. Sec. 1.5 (Validation of the compacted de Bruijn graphs):

We had also manually investigated some output compacted dBGs from these tools for small reference se- quences (the k-mer content is the same in this case). For some given sequence, let the cuttlefish unitigs be Uc and those from BCALM / Bifrost be Ub . We observed that for unitigs u ∈ Ub − Uc , u is present as a substring of some unitig u ′ ∈ Uc − Ub. In the other direction, for unitigs u ∈ Uc − Ub, there exist unitigs u1, u2 ∈ Ub – Uc such that u = u1 ⊙ u2. This conforms to the reasoning that we have added to the paper.

Second, on page 5 line 33, the authors state “BCALM 2 also has a maximum-memory option, which we set to the best memory usage obtained from the rest of the algorithms.” Is there a reason here not to use the max memory setting to that actually used by cuttlefish, for direct comparison? It should be straightforward to show that bcalm can work within the same memory limits as cuttlefish, but slower.

The best memory usage obtained from the rest of the algorithms has always been the one from that of Cuttlefish 2 (we did not exclude Cuttlefish from consideration when stating ‘’rest’’ of the algorithms). Note that, although we set BCALM 2’s memory this way, it did not necessarily conform to the bound for most of the datasets. We had reached out to the BCALM 2 authors on this issue, and the lead and corresponding author had confirmed that they also had similar experiences—the current behavior of BCALM 2 does not necessarily obey this bound. Specifically, they executed BCALM 2 on the SRX016231 human dataset that had been originally used in the BCALM 2 publication, and noted that it incurred 7+ GB memory, even with the setting -max-memory 3000, i.e. < 3 GB. This conforms to our experience of BCALM 2 on the similar HG004 human dataset, where BCALM2 used 6.7—10.5 GB memory, with -max-memory set to 3.9—4.5 GB. They consider that this could be due to the various speed-memory performance tradeoff optimizations implemented since the original publication.

Third, we think an experiment using an RNA-seq dataset might be relevant as it has a different k-mer spectra, and removing errors with a minimum abundance threshold is not as effective as with DNA samples.

Experiment results for all the tested tools have been added into the manuscript for an RNA-seq dataset in Sec. 2.3 (Compacted graph construction for sequencing data).

Minor comments:

* command lines are provided in the supplementary materials but without the actual parameter values. The authors should make their benchmarking scripts available.

* there is a typo in Figure 2 and Supplementary Figure 2: ‘contigs extraction’ should be ‘unitigs extraction’.

* on page 11 column2, Sv is defined as the side of vertex v, while S is defined as a string, and S1 is the first character in the string. We recommend using a different letter for the side of vertex.

* on page 12 line 59, the reference [75] is misleading. The text is talking about piecewise graph traversal, but the reference seems to be about graph traversal techniques?

* the authors described how they calculated f0 (minimum abundance) in the supplementary material but they didn’t provide the actual values used by each experiment.

* in table 2 and 3, we noticed that CUTTLEFISH2 unrestricted memory mode uses two or more times the memory used in the default memory parameters, while the run time is approximately the same. So, what would motivate a user to go for the unrestricted memory mode?

• The benchmarking scripts used in the experiments have been made available in the GitHub repository

https://github.com/COMBINE-lab/cuttlefish_experiments, and it has been referred to in the Suppl.

Sec. 1.10 (Tools and execution commands).

• We have fixed the figure legends in the parallel-scaling plots.

• We have replaced the notation of the side of a vertex with ψ, from s.

• The reference to Kleinberg J and Tardos E (2005) has been made in the context of referring to typical graph traversal algorithms, e.g. the Breadth-first and the Depth-first search procedures, and their potential usage in constructing the compacted de Bruijn graph given the regular graph.

• All the frequency thresholds f0 are mentioned in the describing text of the corresponding tables, i.e. in their sub-captions.

• We believe that this is due to the disk characteristics—all of our experiments have been performed on a few typical hard drives. The most IO-bound step in the algorithm is the enumeration of the edge set, i.e. (k + 1)-mers enumeration, while rest of the steps also iterate through the in-disk vertex or edge databases. So it could be reasonable to expect better timing-profile of the tool, when executing on faster external storages, e.g. SSD or NVMe drives. This is evidenced by Kokot et al [2], who showed that KMC 3 can have considerable gains in speed on large datasets when executed on SATA SSDs. Unfortunately, we do not have any such external disks in our disposal currently, and consequently present this as a hypothesis without results.

Bibliography

[1] Guillaume Rizk, Dominique Lavenier, Rayan Chikhi, DSK: k-mer counting with very low memory usage,

Bioinformatics, Volume 29, Issue 5, 1 March 2013, Pages 652–653, https://doi.org/10.1093/bioinformatics/btt020.

[2] Marek Kokot, Maciej Długosz, Sebastian Deorowicz, KMC 3: counting and manipulating k-mer statistics,

Bioinformatics, Volume 33, Issue 17, 01 September 2017, Pages 2759–2761, <https://doi.org/10.1093/bioinformatics/btx304>.

**2^nd^ round**

**Reviewer 1**

The authors have resolved all my previous concerns and I don't have further comments. Congrats to the authors!

**Reviewer 2**

We appreciate the revisions! Thank you!
